# Disruptions of Anaerobic Gut Bacteria Are Associated with Stroke and Post-stroke Infection: a Prospective Case–Control Study

**DOI:** 10.1007/s12975-020-00863-4

**Published:** 2020-10-14

**Authors:** Bastiaan W. Haak, Willeke F. Westendorp, Tjitske S. R. van Engelen, Xanthe Brands, Matthijs C. Brouwer, Jan-Dirk Vermeij, Floor Hugenholtz, Aswin Verhoeven, Rico J. Derks, Martin Giera, Paul J. Nederkoorn, Willem M. de Vos, Diederik van de Beek, W. Joost Wiersinga

**Affiliations:** 1 Center for Experimental and Molecular Medicine, Amsterdam Infection & Immunity Institute, Amsterdam UMC, location AMC,, University of Amsterdam, Meibergdreef 9, Amsterdam, The Netherlands; 2 Department of Neurology, Amsterdam Neuroscience, Amsterdam UMC, location AMC,, University of Amsterdam, Meibergdreef 9, 1105 AZ Amsterdam, the Netherlands; 3 Center for Proteomics and Metabolomics, Leiden University Medical Center, Leiden, The Netherlands; 4 Laboratory of Microbiology, Wageningen University, Wageningen, The Netherlands; 5 Human Microbiome Research Program, Faculty of Medicine, Helsinki University, Helsinski, Finland; 6 Department of Medicine, Division of Infectious Diseases, Amsterdam Infection & Immunity Institute, Amsterdam UMC, location AMC, University of Amsterdam, Meibergdreef 9, Amsterdam, The Netherlands

**Keywords:** Stroke, Microbiome, Butyrate, Trimethylamine-N-oxide

## Abstract

In recent years, preclinical studies have illustrated the potential role of intestinal bacterial composition in the risk of stroke and post-stroke infections. The results of these studies suggest that bacteria capable of producing volatile metabolites, including trimethylamine-N-oxide (TMAO) and butyrate, play opposing, yet important roles in the cascade of events leading to stroke. However, no large-scale studies have been undertaken to determine the abundance of these bacterial communities in stroke patients and to assess the impact of disrupted compositions of the intestinal microbiota on patient outcomes. In this prospective case–control study, rectal swabs from 349 ischemic and hemorrhagic stroke patients (median age, 71 years; IQR: 67–75) were collected within 24 h of hospital admission. Samples were subjected to 16S rRNA amplicon sequencing and subsequently compared with samples obtained from 51 outpatient age- and sex-matched controls (median age, 72 years; IQR, 62–80) with similar cardiovascular risk profiles but without active signs of stroke. Plasma protein biomarkers were analyzed using a combination of nuclear magnetic resonance (NMR) spectroscopy and liquid chromatography–mass spectrometry (LC-MS). Alpha and beta diversity analyses revealed higher disruption of intestinal communities during ischemic and hemorrhagic stroke compared with non-stroke matched control subjects. Additionally, we observed an enrichment of bacteria implicated in TMAO production and a loss of butyrate-producing bacteria. Stroke patients displayed two-fold lower plasma levels of TMAO than controls (median 1.97 vs 4.03 μM, Wilcoxon *p* < 0.0001). Finally, lower abundance of butyrate-producing bacteria within 24 h of hospital admission was an independent predictor of enhanced risk of post-stroke infection (odds ratio 0.77, *p* = 0.005), but not of mortality or functional patient outcome. In conclusion, aberrations in trimethylamine- and butyrate-producing gut bacteria are associated with stroke and stroke-associated infections.

**Electronic supplementary material:**

The online version of this article (10.1007/s12975-020-00863-4) contains supplementary material, which is available to authorized users.

## Introduction

Acute ischaemic stroke and intracerebral hemorrhage affect millions of people annually across the world. Stroke patients have shown to display increased susceptibility to infections, most commonly pneumonia and urinary tract infections, which has been associated with unfavorable clinical outcome and death [1]. The increased susceptibility to infections following stroke has been suggested to result from activation of long-distance feedback loops between the central nervous system and peripheral immune organs [2], yet stroke-induced intestinal hyperpermeability and dissemination of residential gut bacteria have recently been implicated in the pathogenesis of post-stroke infections [3]. A recent randomized clinical trial investigated the benefit of preventive administration of broad-spectrum antibiotics following stroke [4]. However, no interventions aimed to decrease post-stroke infections have proven to be effective in improving outcomes following stroke. In fact, it has been recently reported that off-target antibiotic disruption of residential microbial communities in the gastrointestinal tract—the intestinal microbiota—may actually worsen stroke outcomes [5, 6].

The microbiota plays a fundamental role on the development and function of the host immune system [7], and an increasing amount of evidence suggests that depletion or enrichment of specific gut bacteria and microbiota-derived metabolites are associated with the pathogenesis of a wide variety of extra-intestinal disorders [5, 8, 9]. This notion is exemplified by trimethylamine (TMA), a microbial metabolite that is produced by various intestinal bacteria from dietary choline and carnitine [10, 11]. TMA is absorbed via the intestinal epithelium and further oxidized to trimethylamine N-oxide (TMAO), which has been associated with an increased accumulation of macrophage-specific cholesterol in atherosclerosis and enhanced activation of platelets, leading to increased risks of major cardiac events [10–13]. In contrast to the detrimental effects of TMAO, several studies have pointed towards the potentially beneficial effects of short-chain fatty acids (SCFA), which represent the primary products of dietary fiber fermentation by the gut microbiota [14]. These metabolites, which are capable of crossing the blood-brain barrier, have shown to play an important role in the maturation of residential immune (microglial) cells of the brain [15]. In addition, it has been shown that transplantation of SCFA-producing bacteria into mice improved outcomes after stroke, in which it is thought that these metabolites, in particular butyrate, attenuate brain inflammation and improve neurogenesis [16, 17].

The results of these studies suggest that bacteria capable of producing volatile metabolites, including trimethylamine-N-oxide (TMAO) and butyrate, play opposing, yet important roles in the potential incidence and outcome of stroke. However, while several small human observational studies have recently been published on this topic [18–21], no large-scale studies using age- and sex-matched control subjects with similar cardiovascular risk factors have been undertaken to determine the abundance of these bacterial communities in patients with acute ischemic stroke and cerebral hemorrhage. In addition, while our group and others have shown in human cohort studies that the abundance of butyrate-producing bacteria has been identified as protective against a variety of systemic infectious diseases, their role in the incidence of post-stroke infections has yet to be elucidated [22–24].

Based on the aforementioned observations, we hypothesize that patients with acute stroke present with a disturbed composition of the intestinal microbiota, which may alter the risk of post-stroke infections following hospitalization. Therefore, we aimed to (1) characterize the gut microbiome of stroke patients and matched controls in a large-scale study and (2) analyze if aberrations in butyrate-producing bacteria are associated with an increased risk of infectious complications and altered clinical outcome following stroke.

## Methods

### Study Design and Participants

This is a sub-study of the Preventive Antibiotics in Stroke Study (PASS), aiming to investigating the impact of the microbiota on post-stroke infections. The PASS study is a multicenter, prospective, randomized, open-label, masked endpoint trial conducted in the Netherlands (ISRCTN66140176) [4]. This trial assessed the clinical benefit of treatment with ceftriaxone within 24 h following admission in addition to stroke unit care in comparison to standard stroke unit care without preventive antimicrobial therapy in 30 academic and non-academic medical center in the Netherlands. A subset of patients, enrolled in three participating centers within this study (Academic Medical Centre, Amsterdam; Radboud University Medical Centre, Nijmegen; and Albert Schweitzer Hospital, Dordrecht), were eligible for participation in a sub-analysis with the aim of investigating the impact of the microbiota on post-stroke infections. Patients were eligible for inclusion if they were aged 18 years or older, had clinical symptoms of a stroke (ischemic or hemorrhagic), an onset of symptoms less than 24 h, and a score of 1 or more on the National Institutes of Health Stroke Scale (NIHSS). Exclusion criteria were clinical signs of infection on hospital admission requiring antibiotic therapy, use of antimicrobials less than 24 h before admission, pregnancy, hypersensitivity for cephalosporins, previous anaphylaxis for penicillin derivatives, subarachnoid hemorrhage, and imminent death. In addition, healthy non-hospitalized age- and sex-matched controls with similar cardiovascular risk profiles but without active signs of stroke were included on a separate study protocol (ClinicalTrials.gov Identifier: NCT02928367) at the outpatient clinic of the Academic Medical Centre, Amsterdam, The Netherlands. The institutional review board of the Academic Medical Centre approved both study protocols. The study was undertaken according to Good Clinical Practice standards and was independently monitored by the Clinical Research Unit of the Academic Medical Centre, University of Amsterdam. All study participants or their legal representatives provided written informed consent.

### Sample Collection

Upon inclusion, one ESwab (FLOQswab™ in 1 mL of Liquid Amies Elution) was inserted in the rectum of a study participant within 24 h of hospital admission and prior to the administration of prophylactic ceftriaxone (if applicable). Upon collection, swabs were transported to the laboratory, after which the FLOQswabs were vortexed to release fecal matter into the Amies solution, which was subsequently stored at − 80 °C. Ethylenediaminetetraacetic acid (EDTA) blood for plasma biomarker measurements was obtained within 24 h of hospital admission in a nested sub-cohort of 35 stroke patients and all controls.

### Microbiota Sequencing

A detailed description of the sequencing procedure is depicted in the Supporting Information Appendix. In short, DNA was extracted using a repeated bead beating protocol, after which the DNA was purified using the Maxwell RSC Whole Blood DNA Kit. 16S rRNA gene amplicons were generated using a single-step PCR protocol targeting the V3–V4 region. The libraries were sequenced using a MiSeq platform using V3 chemistry with 2 × 251 cycles. Amplified Sequence Variants (ASVs) were inferred for each sample individually with a minimum abundance of 4 reads. Unfiltered reads were then mapped against the collective ASV set to determine the abundances. Taxonomy was assigned using the RDP classifier and SILVA 16S ribosomal database V132 [25, 26]. Given the potential role TMAO and butyrate in the context of stroke, we aimed to identify the proportion of TMA- and butyrate-producing bacteria in stroke patients and controls based on a two recently published metagenomic overviews (Tables S1 and S2) [27, 28]. We also calculated the ratio of Firmicutes to Bacteroidetes, which has been considered a marker of microbiota disruption and has been associated with obesity, diabetes, and hypertension [29]. Finally, given the known differences between sex and stroke epidemiology, incidence, and outcome [30], we aimed to verify if male and female stroke patients displayed altered alpha and beta diversity metrics.

### Protein Plasma Biomarker and Targeted TMAO Measurements

Plasma (lipo)protein biomarkers were measured in EDTA plasma of a subset of stroke patients and controls using proton nuclear magnetic resonance (^1^H-NMR) spectroscopy, using a 600-MHz Bruker Avance II spectrometer (Bruker BioSpin, Karlsruhe, Germany). Targeted TMAO measurements were performed using a high-performance liquid chromatography system consisting of an Ultimate 3000 Rapid Separation Quaternary System (ThermoFisher Scientific), combined with a maXis impact HD UHR-QqTOF mass spectrometer from Bruker Daltonics (Bremen, Germany). A detailed description of the sample preparation and measurement of EDTA plasma samples is described in the SI Appendix.

### Statistical Analysis

Statistical analysis was performed in the R statistical framework (Version 3.5.1, Vienna, Austria). To assess alpha diversity, we calculated the Shannon Diversity Index, Inverse Simpson Index, and Observed Taxa Richness index with the phyloseq package [31]. Data were not normally distributed and were therefore analyzed using a Wilcoxon rank sum test. We judged two-tailed *p* values less than 0.05 to indicate statistical significance. Beta diversity differences were calculated using Bray Curtis distance metrics, as well as weighted and unweighted UniFrac distance metrics, after which principal coordinates analysis (PCoA) was performed. Differences in microbiota composition among groups and time points were tested for using permutational multivariate analysis of variance (PerMANOVA), using the vegan R package. To identify taxa that may be driving the significant differences detected between groups, differential abundance analysis was determined using DESeq2. Unsupervised *k*-means clustering analysis on plasma protein biomarkers was performed using the package pheatmap. Finally, potential microbiota-dependent predictors of outcome were assessed using multivariate logistic regression with age, sex, history of diabetes, prior stroke, stroke severity, and prophylactic ceftriaxone exposure, as dependent variables, which are known to be the predictors of stroke outcome and risk of post-stroke infections [4, 32]. The primary endpoint was infection rate following stroke, as judged by an independent adjudication committee (masked to treatment allocation) according to modified Centers for Disease Control and Prevention criteria and compliant with the Recommendations From the Pneumonia in Stroke Consensus Group [4, 33]. Secondary endpoints were mortality and functional outcome at 3 months, of which the latter endpoint was defined by the dichotomized modified Rankin Scale (mRS), in which a score of 0–2 as favorable and a score of 3–6 as unfavorable [4].

## Results

Between July 2010 and March 2014, 2550 adult patients from 30 Dutch sites were enrolled and randomly assigned in a 1:1 ratio to the two study groups. Rectal swabs were collected within 24 h of hospital admission and prior to antibiotic administration in a subset of 349 stroke patients (flow diagram in Fig. S1). The median National Institutes of Health Stroke Scale (NIHSS) at admission was 5 (IQR 3–9), and intravenous thrombolytic therapy was given to 115 (33%) of 349 patients. The definite diagnosis was assessed at discharge in 349 patients: 287 (82%) had ischemic stroke (IS), 25 (7%) had a transient ischemic attack (TIA), and 37 (11%) were diagnosed with a cerebral hemorrhage (CH). About 186 stroke patients included in the sub study (54.9%) received prophylactic ceftriaxone treatment within 24 h of hospital admission. Fifty-one age- and sex-matched controls were included between October 2017 and March 2018. Demographic and clinical characteristics of the subset of patients included for microbiota analysis were comparable to the total case mix of included patients (Table S3). Demographic characteristics and comorbidities between stroke patients and controls were similar (Table 1), although patients with stroke had a higher prevalence of prior stroke (*p* < 0.001) and tobacco addiction (*p* = 0.015) compared with controls, while non-stroke controls had a higher incidence of malignancy (p < 0.001).

**Table 1:** Baseline characteristics

	Control (*n* = 51)	Stroke patient (*n* = 349)	*p*
Age, years	71 [67–75]	72 [62–80]	0.743
Male sex	29 (56.9)	194 (55.6)	0.984
Caucasian ethnicity	48 (94.1)	314 (90.2)	0.789
History			
Atrial fibrillation/flutter	4 (7.8)	56 (16.0)	0.186
Prior stroke	3 (5.9)	109 (31.2)	<0.001
Hypertension	25 (49.0)	208 (59.6)	0.201
Myocardial infarction	10 (19.6)	44 (12.6)	0.251
Cardiac valve disease†	1 (2.0)	25 (6.9)	0.294
Peripheral vascular disease	2 (3.9)	24 (6.3)	0.717
COPD	4 (7.8)	30 (8.6)	1.000
Diabetes mellitus	7 (13.7)	67 (19.2)	0.455
Malignancy	15 (29.4)	31 (8.6)	<0.001
Current smoker	6 (11.8)	104 (29)	0.015
Alcoholism	2 (3.9)	21 (6.0)	0.781
Previous medication			
Anticoagulants	5 (9.8)	37 (10.6)	1.000
Antiplatelet therapy	19 (37.3)	132 (37.8)	1.000
Statins	18 (35.3)	134 (38.4)	0.786
Angiotensin-converting enzyme inhibitors	12 (23.5)	112 (32.5)	0.261
Proton pump inhibitors	20 (39.2)	94 (26.9)	0.099
β-blocker	14 (27.5)	123 (35.2)	0.349
Randomization to Ceftriaxone		186 (54.9)	
Stroke characteristics			
Cerebral infarction		287 (82.2)	
Transient ischemic attack		25 (7.2)	
Cerebral hemorrhage		37 (10.6)	
Modified Rankin Scale score before stroke symptoms$		0 (0–1)	
National Institutes of Health Stroke scale score¶		5 (3–9)	
Dysphagia		96 (27.5)	
Unfavorable outcome		124 (35.5)	
90-day mortality		41 (11.7)	

Data are median (IQR) or *n*/*N* (%). †Cardiac valve disease was defined as cardiac valve insufficiency, stenosis, or replacement. §Scores on the modified Rankin Scale range from 0 to 6, with 6 indicating death; modified Rankin Scale scores before onset of stroke symptoms were assessed in 345 stroke patients. ¶Scores on the National Institutes of Health Stroke Scale range from 0 to 30, with 30 indicating highest degree of stroke severity; these scores were assessed in all 349 patients

### Altered Microbiota Composition in Patients with Stroke

**Fig. 1:**
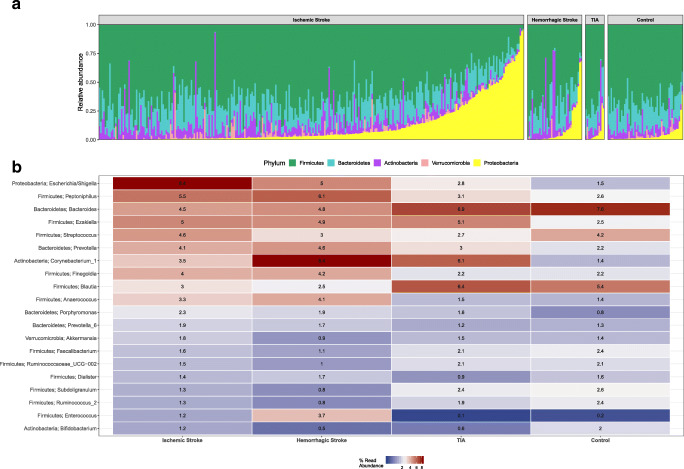
Fecal microbiota composition and alpha diversity among stroke patients and controls. **a** Each bar represents one sample; phyla are indicated with colors and expressed in percentage of the total DNA reads. Only phyla that made up ≥ 5 of the total microbiota in at least one sample are included. On microbiota phylum level, ischemic stroke patients (*n* = 287) and cerebral hemorrhage (*n* = 37) showed a significant reduction in Firmicutes and Bacteroidetes, while Proteobacteria were enriched, compared with controls (*n* = 51). **b** Heatmap of the 20 most abundant bacterial genera in the dataset. Values and colors depicted in the graph display median relative abundance per group

**Fig. 2:**
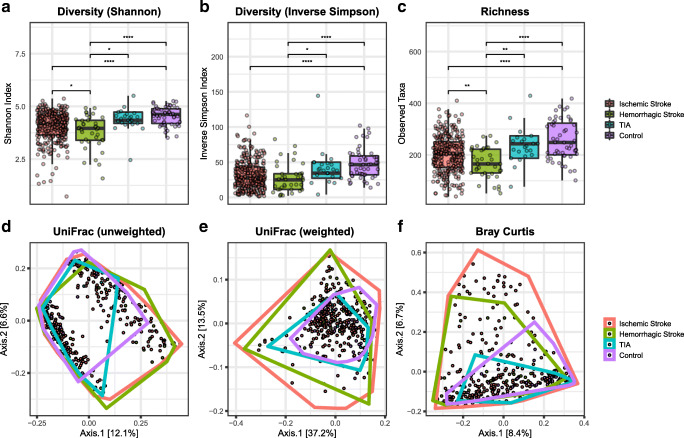
Alpha and beta-diversity differences between stroke patients and controls. The Shannon index (**a**), Inverse Simpson Index (**b**), and the Observed Taxa (**c**) index were used to calculate the alpha diversity community and richness within each individual microbiota sample. Data are presented as box plot overlaid by a dot plot with a line at the median. Beta diversity as depicted by unweighted UniFrac (**d**), weighted UniFrac (**e**), and Bray-Curtis dissimilarity index (**f**) in a PCoA representation. *P < 0.05; **P < 0.01; ***P < 0.001; ****P < 0.0001

Sequencing of fecal samples yielded a total of 21,116,277 high-quality 16S rRNA gene sequences (average 52,791 per sample). Gut microbiota composition of patients with IS and CH was altered compared with matched controls, whereas patients with a TIA displayed comparable microbiota composition. On a microbiota phylum level, IS and CH patients displayed a reduction in Firmicutes and Bacteroidetes, while Proteobacteria were enriched compared with matched controls (Fig. 1a). On a genus level, patients with IS an CH displayed elevated levels of *Escherichia/Shigella*, *Peptoniphilus*, *Ezakiella*, and *Enterococcus*, while controls and patients with a TIA displayed higher abundance of *Blautia*, *Subdoligranulum*, and *Bacteroides* (Fig. 1b). These findings were confirmed by Benjamini–Hochberg corrected DESEq2 analysis, which showed a strong decrease in obligate anaerobic genera, such as *Anaerostipes*, *Ruminococcus*, and *Subdoligranulum*, while potentially invasive aerobic bacterial genera, including *Enterococcus* species and *Escherichia/Shigella* species, were enriched in all stroke patients (Fig. S2). In tandem with the differences in community composition, strong alterations were observed in alpha diversity and richness in patients with IS and CH compared with controls (median Shannon index: IS 4.34 and CH 4.05 vs Control 4.65; Wilcoxon *p* < 0.0001 and *p* < 0.0001, respectively; median Inverse Simpson index: IS 28.7 and CH 25.2 vs Control 48.7, Wilcoxon *p* < 0.0001 and *p* < 0.0001, respectively; median Observed Taxa: IS 205 and CH 163 vs Control 249, Wilcoxon *p* < 0.0001 and *p* < 0.0001, respectively; Fig. 2a–c). Similar patterns were observed in beta diversity metrics of the microbiota, as samples that were collected from IS and CH patients had a significantly altered Bray-Curtis dissimilarity index, as well as weighted and unweighted UniFrac distance metrics compared with controls (PERMANOVA, *p* < 0.0001 for all comparisons; Fig. 2e–g). In contrast to the observations with IS and CH patients, we did not observe differences in community composition, as well as alpha and beta diversity metrics in TIA patients compared with controls. In addition, we did not observe any differences in the Firmicutes to Bacteroidetes ratio between stroke patients and controls (Fig. S3). Finally, alpha and beta diversity profiles were comparable between male and female stroke patients (Fig. S4).

### Stroke Patients Display a Higher Prevalence of TMA-Producing Bacteria and Lower Plasma Levels of TMAO

**Fig. 3:**
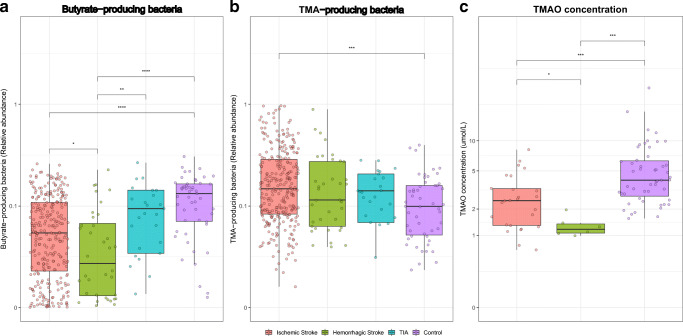
Higher amounts of trimethylamine-producing bacteria with low abundance of trimethylamine-N-oxide in fecal samples of ischemic stroke patients. Abundance of butyrate-producing bacteria (a), TMA-producing bacteria (**b**), and absolute TMAO concentration (**c**), in fecal samples of patients with stroke or healthy controls. Data are presented as box plot overlaid by a dot plot with a line at the median. *P < 0.05; **P < 0.01; ***P < 0.001; ****P < 0.0001

Next, given the preclinical observations implicating TMA- and butyrate-producing bacteria as important mediators of cardiovascular events and stroke outcome, respectively, we aimed to identify overall abundance of these groups of bacteria (depicted in Table S1–2) in our cohort. First, we observed that IS and CH patients displayed significantly lower abundances of butyrate-producing bacteria compared with TIA patients and controls. In addition, IS patients, but not patients with CH or TIA, harbored elevated levels of TMA-producing genera (median abundance IS 0.178 vs Control 0.101, Wilcoxon *p* < 0.0001; Fig. 3b). Patients with severe stroke, defined as a NIHSS > 10, had a higher abundance of TMA-producing bacteria compared with patients with less severe stroke (median abundance 0.141 vs 0.196, Wilcoxon *p* = 0.023; Fig. S5A). To validate these findings, we performed a combination of nuclear magnetic resonance (NMR) spectroscopy and liquid chromatography–mass spectrometry (LC-MS) to measure (lipo)protein biomarkers, and TMAO, in plasma collected in 35 patients and 51 controls. Baseline characteristics of the patients included and not-included in this subset were comparable (Table S4). Lipoprotein levels between patients and controls were similar (Table S5). Unsupervised *k*-means clustering analysis of 30 plasma metabolites revealed a clustering of samples collected in patients with stroke compared with non-stroke controls (Fig. S6). Clustering was driven by higher levels of glucose, acetone, and β-hydroxybutyrate, and lower levels of dimethylamine, glutamine and methanol in stroke patients as compared with controls (Fig. S7), which is in line with previous NMR analyses during stroke [34]. Plasma concentrations of TMAO were two-fold lower in stroke patients compared with controls (median 1.97 vs 4.03 μM, Wilcoxon *p* < 0.0001; Fig. 3b) In addition, plasma concentration of TMAO did not correlate with the abundance of TMA-producing bacteria, regardless of stroke severity (Figure S5B).

### Abundance of Intestinal Butyrate-Producing Bacteria Is an Independent Predictor of Post-stroke Infections

**Fig. 4:**
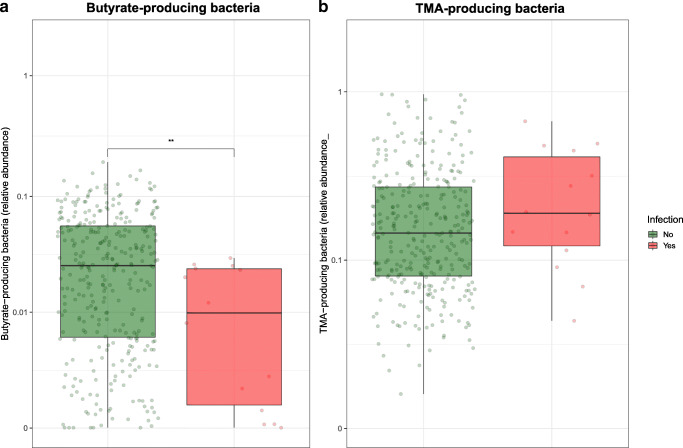
Comparison of butyrate- and TMA-producing bacteria in patients with post-stroke infections. Data are presented as box plot overlaid by a dot plot with a line at the median. **P < 0.01

Outcome was evaluated in all 349 patients: 124 (35.5%) had an unfavorable outcome, including 41 (11.7%) who died within 3 months after admission. The adjudication committee (expert panel) diagnosed post-stroke infection in 14 (4%) of 349 patients during hospital stay: urinary tract infection in 9 patients (3%), pneumonia in 4 patients (1%), and other infections in one patient (< 1%).Preventive ceftriaxone did not result an altered alpha diversity compared with standard stroke care (Fig. S8). Patients with post-stroke infection displayed lower abundance of butyrate-producing bacteria compared to patients that did not develop post-stroke infections (media abundance, 0.045 vs 0.009; Wilcoxon *p* = 0.002), while no differences in TMA-producing bacteria were detected between groups (Fig. 4). Logistic multivariate regression analysis indicated that every log increase in the abundance of butyrate-producing bacteria was associated with a decreased risk of developing post-stroke infections, independent of age, sex, history of diabetes, prophylactic ceftriaxone treatment, prior stroke, and stroke severity (Odds Ratio 0.74, multivariate *p* = 0.005; Table 2). These findings remained significant in a separate sensitivity analysis where patients receiving prophylactic ceftriaxone treatment were excluded (Table S7). Higher abundance of butyrate-producing bacteria was associated with favorable outcome, independent of age, sex, history of diabetes, ceftriaxone treatment, and prior stroke. However, this association did not remain robust with the inclusion of stroke severity into the multivariate model. Taken together, these findings indicate that IC and CH patients display aberrations in the composition of the intestinal microbiota, which is associated with an increased risk of stroke-associated infections in the days following hospital admission.

**Table 2:** Logistic regression on role of butyrate-producing bacteria on stroke outcome

	Univariate		Multivariate	
	Odds ratio (2.5–97.5%)	*p* value	Odds ratio (2.5–97.5%)	*p* value
Clinical infection				
Age	1.04 [1.00–1.09]	0.10		
Male sex	1.07 [0.36–3.31]	0.905		
Diabetes	1.73 [0.46–5.35]	0.369		
Prior stroke	0.88 [0.24–2.69]	0.827		
NIHSS > 10	3.65 [1.21–11.00]	0.019	3.07 [0.94–9.87]	0.058
Butyrate-producing bacteria (log abundance)	0.74 [0.61–0.90]	0.001	0.74 [0.60–0.91]	0.005
Ceftriaxone exposure	0.21 [0.05–0.67]	0.017	0.16 [0.03–0.56]	0.009
90-day mortality				
Age	1.07 [1.04–1.11]	<0.001	1.06 [1.03–1.10]	<0.001
Male sex	0.59 [0.30–1.13]	0.112		
Diabetes	1.65 [0.75–3.41]	0.190		
Prior stroke	1.31 [0.65–2.57]	0.432		
NIHSS > 10	8.34 [4.19–17.17]	<0.001	8.27 [4.03–17.52]	<0.001
Butyrate-producing bacteria (log abundance)	0.91 [0.79–1.06]	0.204		
Ceftriaxone exposure	0.21 [0.05–0.67]	0.160		
Unfavorable outcome*				
Age	1.04 [1.02–1.06]	< 0.001	1.04 [1.02–1.06]	< 0.001
Male sex	0.47 [0.23–0.73]	<0.001	0.67 [0.40 – 1.12]	0.129
Diabetes	1.77 [1.03–3.04]	0.038	2.15 [1.14 – 3.76]	0.016
Prior stroke	1.49 [0.93–2.37]	0.093		
NIHSS > 10	9.38 [5.32–1 .20]	< 0.001	9.88 [5.32–19.20]	< 0.001
Butyrate-producing bacteria (log abundance)	0.88 [0.78–0.98]	0.043	0.89 [0.7–1.02]	0.266
Ceftriaxone exposure	1.15 [0.60–2.27]	0.675		

## Discussion

This observational case–control study shows that stroke is associated with significant disturbances of gut bacterial profiles, specifically in the context of ischemic stroke and cerebral hemorrhage. We showed that these patients had lower microbiota richness, alpha diversity, and lower amounts of intestinal anaerobic bacteria as well as a higher prevalence of potentially pathogenic bacterial taxa compared with patients with TIA and matched controls with similar cardiovascular risk factors. Currently, four pilot studies have been published on the composition of the microbiota in human patients with stroke, although presentation of these disruptions appears to be heterogeneous. Similar to our findings, two studies observed that the intestine of stroke patients appears to be overgrown with Proteobacteria with a simultaneous loss of obligate anaerobic bacteria, [19, 20] whereas two studies did not observe differences between groups [18, 21]. Given the notion that current data is derived from small groups of patients, we aimed to analyze the gut microbiota of a well-characterized subpopulation of stroke patients included in a pragmatic randomized clinical study, [4] and compared results with age- and sex-matched control subjects with similar cardiovascular risk factors and plasma lipoprotein levels. As preclinical observations have implied that TMAO and TMA-producing bacteria are associated with cardiovascular events [19, 35], we aimed to evaluate the abundance of the major producers of this metabolite in our cohort. Indeed, we observed that patients with ischemic stroke displayed substantially larger amounts of gut bacteria that harbored key TMA-synthesis genes in a stroke-severity dependent manner, indicating that these micro-organisms could indeed be of importance in the onset of stroke. These findings are in line with preclinical and clinical studies describing that consumption of a high-fat and carnitine-rich diet raises the abundance of TMA-producing gut bacteria, which are thought to promote TMAO-induced platelet activation with enhanced risk of thrombotic events [10–13]. This concept has been reinforced by murine models that have shown that TMAO-induced thrombosis risk is transmissible by microbiota transplantation [12]. Our findings might imply that disruptions of the microbiome may precede the occurrence of the disease, and these disruptions are therefore potentially contributing to an increased risk of both ischemic stroke and cerebral hemorrhage [12, 13]. These observations are supported by a recent study, which showed that patients with highest risk of developing stroke displayed larger disruptions of the microbiota, indicating that changes in the microbiota precede the development of stroke [36]. In turn, stroke itself can alter the intestinal microbiota, which is underscored by murine studies that show that traumatic events, including occlusion of cerebral arteries, can lead to increased intestinal hyperpermeability and an altered intestinal ecosystem [3]. Longitudinal cohort analyses are warranted to further confirm a causal and bidirectional link between disruptions of intestinal communities and the cardiovascular events in the brain. The observation that patients with both ischemic and hemorrhagic stroke display similar disruptions of the microbiome could be based on the notion that these two presentations of disease, while fundamentally different, are intrinsically linked by similar predisposing conditions and risk factors [37–39]. Of note, we observed that patients with hemorrhagic stroke and patients with high NIHS scores, displayed stronger disruptions compared with ischemic stroke patients and patients with a transient ischemic attack, which is in line with previous studies that the degree of microbiota disruption correlates positively with stroke severity [20, 40].

To our surprise, we observed that high levels of TMA-producing gut bacteria did not correspond with elevated TMAO levels in plasma, as stroke patients had lower levels of the metabolite in the acute phase of the disease. However, our findings overlap with similar cross-sectional observations [19] as well as a recent longitudional studies showing that TMAO levels decrease in the hours and days following stroke [40, 41]. Despite of an increased understanding of the impact of TMAO on thrombosis potential, it remains unknown how TMAO specifically interacts with platelets. However, it is likely that iatrogenic factors, such as treatment with intravenous thrombolytic therapy, could alter TMAO levels in the acute phase of stroke [40]. In addition, intrinsic pathophysiological mechanisms of stroke could contribute to lower TMAO levels, as the compound possesses both hydrophobic and hydrophilic properties, and studies with isotope-labeled TMAO have shown that the compound has the potential to enter cells [12]. Therefore, the low plasma levels of TMAO we observed could potentially be explained by either enhanced use or increased intracellular presence of TMAO in the acute phase of stroke, rendering it undetectable in the systemic circulation. Further insight into the properties, pharmacokinetics, and mechanisms of action of this metabolite remain of paramount interest.

Recent studies have uncovered significant insights into the immunomodulatory role of SCFAs, both in the systemic circulation and in the brain, which could play a role in stroke outcome [16]. Therefore, we aimed to observe if patients with stroke displayed altered abundances of obligately anaerobic bacteria capable of producing these metabolites. We specifically focused on butyrate-producing bacteria, given observations in earlier cohort studies that these bacteria have shown to be independently associated with a reduced risk of the development of infections [24, 42]. In line with earlier findings, we observed that lower abundance of anaerobic gut bacteria with butyrate-producing potential was an independent predictor of post-stroke infections, further underscoring the importance of these organisms in the resistance against infections. It has been shown that higher fecal presence and intravenous administration of butyrate has the capacity to enhance the antimicrobial activity of monocytes and macrophages in vitro*,* [23] as well as directly provide antimicrobial resistance against respiratory pathogens in vivo [22, 24]. The immunomodulatory capacity of butyrate has been attributed to its potential to inhibit histone deacetylases and mTOR signaling within circulating leukocytes, rendering these capable of providing a balanced host response to invading pathogens [22, 23, 43]. However, while butyrate is an important element in the immunomodulatory arsenal of the intestinal microbiota, it is important to realize that butyrate-producing bacteria are capable of producing a wide range of other metabolites, such as other SCFAs, indoles, and desaminotyrosine, which are also are able to directly contribute to resistance against infections. Therefore, intestinal presence of butyrate-producing bacteria could be an uncharted marker of immunological resilience that extends beyond the effects of butyrate alone [44]. Preclinical murine research has shown that targeted depletion of obligate anaerobic bacteria are associated with increased post-stroke inflammation in a T helper cell 17-dependent manner, leading to decreased functional outcome in mice, [5] and univariate analysis within our cohort revealed that butyrate-producing bacteria indeed display a role in altered stroke outcomes. This notion has been supported by animal models of stroke that have demonstrated that T regulatory cells, which are upregulated in response to the presence of butyrate-producing bacteria, [45] contribute to the dampening of post-ischemic inflammation of resident and invading inflammatory cells [16, 46]. However, inclusion of stroke severity in the model negated the protective effect of these communities, underscoring that the intrinsic complexity of the disease impedes straightforward translation of murine results into practical microbiota-targeted applications for stroke patients.

This study has several limitations. First, the cross-sectional design of our study limited our ability to provide clear insights on both the causes and long-term consequences of microbiota disruptions during stroke. Second, we were not able to determine the levels of short-chain fatty acids in this cohort, as the medium that was used in the rectal swab collection tubes impeded us to do metabolomic analyses. Nevertheless, we have validated the relationship between the abundance of these butyrate-producing bacteria and the absolute concentrations of all SCFAs in recent publications [24, 47] and are therefore confident that an absence of these bacteria leads to intestinal depletion of these metabolites.

Third, we used patients included in a randomized study on preventive antibiotics [4], which could have implications for the outcome measurements of this study. However, we accounted for prophylactic ceftriaxone treatment in separate sensitivity analysis, justifying the inclusion of both treatment groups. Fourth, while the PASS study had excellent characterization of patients and their outcome, including infections, this will inevitably lead to selection bias. Fifth, the incidence of infection and the number of patients that provided plasma samples in this study were low, which hampered the power of our analyses. The relatively low number of infections could be explained by the inclusion of mild stroke patients with low NIHSS scores, which tend to be less susceptible to infection [4, 48]. Finally, despite our efforts to address confounding—by using an age-matched cohort with comparable demographic characteristics and comorbidities—the described differences between stroke patients and matched controls might reflect undocumented differences, such as dietary changes, prior antibiotic exposure, and infarct etiologies, between these groups as well. For example, around 25% of patients had dysphagia at hospital admission and received tube feeds rather than solid food, potentially impacting the microbiome composition of these patients. Further validation of these findings in a larger longitudinal setting is warranted.

In conclusion, the study of the immune system is receiving increasing attention in the field of stroke. We observed altered microbiota composition in adults with acute ischemic stroke and cerebral hemorrhage compared with age- and sex-matched controls with similar cardiovascular risk profiles and lipoprotein levels. Butyrate-producing bacteria abundance was independently associated with reduced infection rate. This study further fuels the hypothesis of the gut-immune-brain axis and stresses the importance of microbiota research in stroke.

## Electronic supplementary material

ESM 1 (DOCX 2916 kb)

## Data Availability

The data and code that support the findings of this study are available from the corresponding author, upon reasonable request.
