# Supplementary material for: Disruptions of Anaerobic Gut Bacteria Are Associated with Stroke and Post-stroke Infection: a Prospective Case–Control Study
Source: Transl Stroke Res. 2020 Oct 14;12(4):581–92. doi: 10.1007/s12975-020-00863-4 (PMC8213601; doi:10.1007/s12975-020-00863-4)
Supplement: Supplementary file 1 — (DOCX 2916 kb) [file 12975_2020_863_MOESM1_ESM.docx]

**Supplemental Material**

**Disruptions of anaerobic gut bacteria are associated with stroke and post-stroke infection:**

**a prospective case-control study**

Bastiaan W. Haak^1^, Willeke F. Westendorp^2^, Tjitske S.R. van Engelen^1^, Xanthe Brands^1^, Matthijs C. Brouwer^2^, Jan-Dirk Vermeij^2^, Floor Hugenholtz^1^, Aswin Verhoeven^3^, Rico J. Derks^3^, Martin Giera^3^, Paul J. Nederkoorn^2^, Willem M. de Vos^4,5^, Diederik van de Beek^2^*, W. Joost Wiersinga^1,6^*

^1^Amsterdam UMC, location AMC, University of Amsterdam, Center for Experimental and Molecular Medicine, Amsterdam Infection & Immunity Institute, Meibergdreef 9, Amsterdam, The Netherlands

^2^Amsterdam UMC, location AMC, University of Amsterdam, Department of Neurology, Amsterdam Neuroscience, Meibergdreef 9, Amsterdam, The Netherlands

^3^Center for Proteomics and Metabolomics. Leiden University Medical Center, Leiden, The Netherlands

^4^Laboratory of Microbiology, Wageningen University, Wageningen, The Netherlands

^5^Human Microbiome Research Program, Faculty of Medicine, Helsinki University, Helsinski, Finland

^6^Amsterdam UMC, location AMC, University of Amsterdam, Department of Medicine, Division of Infectious Diseases, Amsterdam Infection & Immunity Institute, Meibergdreef 9, Amsterdam, The Netherlands

*Shared last authors

Corresponding author: Diederik van de Beek, MD, PhD, Amsterdam UMC, location AMC, Department of Neurology, Amsterdam Neuroscience, Meibergdreef 9, Amsterdam, The Netherlands, 1105 AZ, Amsterdam, the Netherlands, Phone: +31-20-5669111, E-mail: d.vandebeek@amsterdamumc.nl

| Participating centers and investigators | 3 |
| --- | --- |
| 16S rRNA sequencing | 4 |
| Nuclear Magnetic Resonance spectroscopy analysis | 6 |
| Targeted TMAO measurements | 8 |
| Supplementary table 1 | 10 |
| Supplementary table 2 | 12 |
| Supplementary table 3 | 13 |
| Supplementary table 4 | 14 |
| Supplementary table 5  Supplementary table 6 | 15  15 |
| Supplementary table 7 | 16 |
| Supplementary figure 1 | 17 |
| Supplementary figure 2 | 18 |
| Supplementary figure 3 | 19 |
| Supplementary figure 4 | 20 |
| Supplementary figure 5 | 21 |
| Supplementary figure 6  Supplementary figure 7  Supplementary figure 8 | 22  23  24 |
| References | 25 |
|  |  |

**Table of contents**

**The following centers and investigators participated in the Preventive Antibiotics in Stroke Study:** Academisch Medisch Centrum, Amsterdam: D. van de Beek, P.J. Nederkoorn, W.F. Westendorp, J-D. Vermeij; Albert Schweitzer Ziekenhuis, Dordrecht: H. Kerkhoff, Elles Zock, Ruud P. Kleyweg; Onze Lieve Vrouwe Gasthuis, Amsterdam: J.L.W. Bosboom, V.I.H. Kwa; Kennemer Gasthuis, Haarlem: M. Weisfelt; Slotervaartziekenhuis, Amsterdam: N.D. Kruyt; Amphia Ziekenhuis, Breda: M.J.M. Remmers; Radboud Universitair Medisch Centrum, Nijmegen: E.J. van Dijk; Sint Franciscus Gasthuis, Rotterdam: F. Vermeij; Atrium Medisch Centrum, Heerlen: A. Schreuder; Ziekenhuis Rijnstate, Arnhem: S.E. Vermeer; Medisch Centrum Alkmaar, Alkmaar: R. ten Houten; Erasmus MC, Rotterdam: D.W.J. Dippel; Universitair Medisch Centrum Utrecht, Utrecht: L.J. Kappelle, H.B. van der Worp; Spaarne Ziekenhuis, Hoofddorp: I.S.J. Merkies; HagaZiekenhuis, Den Haag: S.F.T.M. de Bruijn, K.F. de Laat; Medisch Centrum Haaglanden, Den Haag: K. Jellema; Catharina Ziekenhuis, Eindhoven: K. Keizer, M.C. de Rijk, A.J. Vermeij; VU Medisch Centrum, Amsterdam: M.C. Visser; Reinier de Graaf Groep, Delft: L.A.M. Aerden; Martini Ziekenhuis, Groningen: E.S. Schut; Ziekenhuisgroep Twente, Almelo: L.J.A. Reichman; Groene Hart Ziekenhuis, Gouda: K. de Gans; Zaans Medisch Centrum, Zaandam: R.M. van den Berg-Vos; Laurentius Ziekenhuis, Roermond: M.P.J. van Goor; IJselland Ziekenhuis, Capelle aan den IJssel: A.D. Wijnhoud; Westfriesgasthuis, Hoorn: T.C. van der Ree; BovenIJ Ziekenhuis, Amsterdam: M. Janmaat; Orbis Medisch Centrum, Sittard: N. van Orshoven; Bronovo Ziekenhuis, Den Haag: S.M. Manschot.

**Study group:** D. van de Beek, P.J. Nederkoorn, D.W.J. Dippel, M.G.W. Dijkgraaf, T. van der Poll, J.M. Prins, L. Spanjaard, F.H. Vermeij.

**Detailed methodology for 16S rRNA sequencing**

DNA was extracted from the Amies solution using a proteinase K pre-treatment followed by a combination of bead-beating and the Maxwell 16 Tissue LEV Total RNA Purification Kit with STAR (Stool transport and recovery) buffer (Roche, Basel Switzerland) [1,2]. Following centrifugation, 250 µl supernatant was used with the Maxwell RSC Blood DNA kit, and the DNA was eluted in ~60 µl DNAse free water. Twenty nanograms of DNA was used for the amplification of the 16S rRNA gene with V3-V4 341F forward and 805R reverse for 25 cycles. The PCR was performed in a total volume of 30 µl containing 1× HF buffer (Thermo Fisher Scientific, Waltham, MA, USA ), 1 µl dNTP Mix (10 mM; Promega, Leiden, the Netherlands), 1 U of Phusion Green High-Fidelity DNA Polymerase(Thermo Fisher Scientific, Waltham, MA, USA), 500nM of the forward 8-nt sample-specific barcode primer containing the Illumina adapter, pad and link (341F (5’- CCTACGGGNGGCWGCAG-3’) 500nM of reverse 8-nt sample-specific barcode primer containing the Illumina adapter, pad and link (805R (5’ GACTACHVGGGTATCTAATCC-3’)) 20 ng/µl of template DNA and nuclease free water. The amplification program was as follows: initial denaturation at 98°C for 30 s; 25 cycles of denaturation at 98°C for 10 s, annealing at 55°C for 20 s, elongation at 72°C for 90 s; and an extension at 72°C for 10 min [3]. The size of the PCR products (~540 bp) was confirmed by gel electrophoresis using 4 µl of the amplification reaction mixture on a 1% (w/v) agarose gel containing ethidium bromide (AppliChem GmbH, Darmstadt (Germany). PCR products were purified with magnetic beads according to Agencourt AMPure XP PCR Purification protocol of the manufacturer’s instructions using a 96 wells format with Biomex FX (Beckman Coulter, Inc. Brea, CA, USA) and 30 µl Nuclease Free Water (Qiagen GmbH, Hilden Germany), and quantified using Qubit dsDNA BR Assay Kit in combination with FLUOstar OPTIMA (BMG LaBTECH, Ortenberg, Germany). The purified PCR products were mixed in approximately equimolar amounts. Purified amplicon pools were 250 bp paired-end sequenced using Illumina Miseq with 2x251 cycles. (Microbiota Center Amsterdam, The Netherlands)

The sequence reads were analysed as follows. Read pairs with perfect matching forward and reverse barcodes were assigned to their corresponding samples. Forward and reverse reads were truncated to 240 and 210 bases respectively and merged using USEARCH [4]. Merged reads that did not pass the Illumina chastity filter, had an expected error rate higher than 2, or were shorter than 380 bases were filtered. Dada2 (v1.5.2) was used to infer, in parallel, the amplicon sequence variants (ASV), with a minimum abundance of 4 reads [5]. Unfiltered reads were than mapped against the collective ASV set to determine the abundances. Taxonomy was assigned using the RDP classifier and SILVA 16S ribosomal database V132 [6,7]. Performance was evaluated by inclusion of two positive controls consisting of genomic material of 55 different strains.

**Detailed methodology for Nuclear Magnetic Resonance (NMR) spectroscopy analysis**

The EDTA plasma samples were thawed at 4°C and were mixed by inverting the tubes 10 times. Next, 125 μl of plasma was manually transferred to a Ritter 96 deepwell plate. After adding 125 µL 75 mM disodium phosphate buffer in H_2_O/D_2_O (80/20) with a pH of 7.4 containing 6.15 mM NaN3 and 4.64 mM sodium 3-[trimethylsilyl] d4-propionate (Cambridge Isotope Laboratories) and sealing the plate with a capmat, the samples were mixed by inverting 10 times. Using a modified Gilson 215 liquid handler, 195 μl of each sample was transferred into 3mm NMR SampleJet tubes. Subsequently the tubes were closed by inserting POM balls into the caps and transferred to the SampleJet autosampler were they were kept at 6°C while queued for acquisition.

All proton nuclear magnetic resonance (^1^H-NMR) experiments were acquired on a 600 MHz Bruker Avance II spectrometer (Bruker BioSpin, Karlsruhe, Germany) equipped with a 5-mm triple resonance inverse (TCI) cryogenic probe head with Z-gradient system and automatic tuning and matching. A standard 3mm sample of 99.8% methanol-d4 (Bruker Biospin) was used for temperature calibration before the measurements [8]. All experiments were recorded at 310 K. The duration of the π/2 pulses were automatically calibrated for each individual sample using a homonuclear-gated nutation experiment on the locked and shimmed samples after automatic tuning and matching of the probe head [9]. For water suppression, presaturation of the water resonance with an effective field of γB1 = 25 Hz was applied during the relaxation delay and the mixing time of the NOESY1D [10]. Four different NMR pulse sequences were applied to each plasma sample: NOESY1D, CPMG, diffusion edited, and 2D JRES.

The NOESY1D experiment was recorded using the first increment of a NOESY pulse sequence [11] with a relaxation delay of 4 s and a mixing time of 10 ms. A total 16 scans of 98,304 points covering a sweepwidth of 18,029 Hz were recorded after applying 4 dummy scans. A standard 1D Carr-Purcell-Meiboom-Gill (CPMG) pulse sequence with presaturation was used for the acquisition of T2-filtered spectra. A pulse train of 128 refocusing pulses with individual spin echo delays of 0.6 ms was applied resulting in a total T2 filtering delay of 78 ms. A total of 73,728 data points covering a spectral width of 12,019 Hz were collected. Otherwise the parameters were similar to the NOESY1D experiment.

The diffusion edited spectrum [12] was generated with a diffusion time of 120 ms. 16 scans of 98,304 points covering a sweepwidth of 18,029 Hz were recorded. Other parameters were similar to the NOESY1D experiment. J-resolved spectra (JRES) were recorded with a relaxation delay of 2 s with presaturation and 2 scans for each increment in the indirect dimension after 8 dummy scans. A data matrix of 40 × 12,288 data points was collected covering a sweep width of 78 × 10,000 Hz.

The transformation of the time domain data and further processing for binning and the relative quantification of small metabolites was performed in the KIMBLE metabolomics workflow [13]. The lipoprotein distribution prediction method was the commercial Bruker IVDr Lipoprotein Subclass Analysis (B.I.-LISA) platform.

**Detailed methodology for targeted TMAO measurements by LC-MS**

TMAO (98%) was purchased from Sigma Aldrich (Zwijndrecht, The Netherlands) and D9-TMAO (98%) was purchased from Cambridge Isotope Labs (Tewksbury, MA, USA). Water (LC-MS Ultra CHROMASOLV, Riedel de Haen), acetonitrile (LC-MS CHROMASOLV, Riedel de Haen) and. Ammonium acetate (> 99%, Fluka) were purchased from Honeywell Research Chemicals (USA). Calibration curves of TMAO were made in 85:15 v/v acetonitrile:water with D9-TMAO (20 ng/mL) as internal standard. The calibration curve was ranged from 0.5 – 1000 ng/mL TMAO. 5 µL of D9-TMAO (end concentration 20 ng/mL) was added to 50 µL plasma sample. Protein precipitation was done with ice-cold acetonitrile (-30 ˚C) at a ratio of 5:1 v/v (acetonitrile/sample). After adding the ice-cold acetonitrile, the sample was briefly vortexed and stored for 30 minutes in a -30 ˚C freezer. Next, the sample was centrifuged (Eppendorf 5427R, Nijmegen, The Netherlands, Eppendorf Netherlands B.V.) at 18,213x g for 15 minutes at 4 ˚C. The supernatant was transferred to a glass vial for analysis.

The HPLC system consisted of an Ultimate 3000 Rapid Separation Quaternary System (ThermoFisher Scientific). Sample was injected onto a HILIC column (Acquity UPLC BEH HILIC 1.7 μm, 2.1 x 100 mm, Etten-Leur, The Netherlands, Waters) with guard column (Acquity UPLC BEH 1.7 μm, 2.1 x 5 mm, Etten-Leur, The Netherlands, Waters). A gradient of acetonitrile (solvent B) and water containing 10 mM ammonium acetate at pH 6.9 (eluent A) was used for the separation. The applied gradient, with a flow of 400 µL/min, was as follows: 0 min. 95% B, 0.6 min. 95%, 8.6 min. 50% B, 9.6 min. 50% B, 9.7 min. 95% B and 13.7 min. 95% B. The column oven was set to 40 ˚C and the autosampler was set to 5 ˚C. The injection volume was 2 µL. A maXis impact HD UHR-QqTOF mass spectrometer from Bruker Daltonics (Bremen, Germany) was used. The maXis impact HD operated in positive ionization mode and acquired data in the mass range from *m/z* 50 to 500 with a spectra rate of 1 Hz. The capillary was set at 4500 V, the end plate offset at 500 V, the nebulizer gas at 1.6 bar and the dry gas at 6 L/min at 350 °C. All samples where measured in a single sequence. To monitor the quality of the sequence, a pooled sample was made from all samples (QCpool). After every 10th sample injection, a QCpool injection was done to monitor the quality of the sequence. Peak integration and export of the results was done with DataAnalysis version 4.2 (build 383.1) (Bruker Daltonics. Germany). The results where further processed with R version 3.6.1 (https://www.R-project.org).

**Supplementary table 1. Trimethylamine (TMA)-producing bacteria selection**

In order to assess the presence of TMA-producing bacteria in our cohort, we measured the abundance of 50 bacterial genera that are known to harbor key genes of the main TMA-synthesis pathways, encoding choline TMA-lyase (cutC) and carnitine oxygenase (cntA), in accordance with a recently published study that analysed TMA-producing pathways from 10 publicly available data sets [14].

| **Phylum** | **Order** | **Class** | **Family** | **Genus** |
| --- | --- | --- | --- | --- |
| Proteobacteria | Gammaproteobacteria | Aeromonadales | Aeromonadaceae | Aeromonas |
| Firmicutes | Clostridia | Clostridiales | Clostridiaceae 2 | Alkaliphilus |
| Firmicutes | Clostridia | Clostridiales | Clostridiales_Incertae Sedis XI | Anaerococcus |
| Firmicutes | Clostridia | Clostridiales | Clostridiales_Incertae Sedis XI | Anaerosalibacter |
| Actinobacteria | Actinobacteria | Coriobacteriales | Coriobacteriaceae | Atopobium |
| Firmicutes | Bacilli | Bacillales | Bacillaceae 1 | Bacillus |
| Proteobacteria | Gammaproteobacteria | Enterobacteriales | Enterobacteriaceae | Brenneria |
| Proteobacteria | Gammaproteobacteria | Enterobacteriales | Enterobacteriaceae | Citrobacter |
| Firmicutes | Clostridia | Clostridiales | Clostridiaceae 1 | Clostridium sensu stricto |
| Firmicutes | Clostridia | Clostridiales | Peptostreptococcaceae | Clostridium XI |
| Firmicutes | Clostridia | Clostridiales | Incertae Sedis XI | Clostridium XII |
| Firmicutes | Clostridia | Clostridiales | Lachnospiraceae | Clostridium XlVa |
| Actinobacteria | Actinobacteria | Coriobacteriales | Coriobacteriaceae | Collinsella |
| Proteobacteria | Gammaproteobacteria | Enterobacteriales | Enterobacteriaceae | Cronobacter |
| Firmicutes | Clostridia | Clostridiales | Peptococcaceae 1 | Desulfitobacterium |
| Proteobacteria | Deltaproteobacteria | Desulfobacterales | Desulfobulbaceae | Desulfobulbus |
| Firmicutes | Clostridia | Clostridiales | Peptococcaceae 1 | Desulfosporosinus |
| Proteobacteria | Deltaproteobacteria | Desulfobacterales | Desulfobulbaceae | Desulfotalea |
| Firmicutes | Clostridia | Clostridiales | Peptococcaceae 2 | Desulfotomaculum |
| Proteobacteria | Deltaproteobacteria | Desulfovibrionales | Desulfovibrionaceae | Desulfovibrio |
| Proteobacteria | delta/epsilonsubdivisions | Deltaproteobacteria | Desulfovibrionales | Desulfovibrionaceae |
| Proteobacteria | Gammaproteobacteria | Enterobacteriales | Enterobacteriaceae | Enterobacter |
| Firmicutes | Bacilli | Lactobacillales | Enterococcaceae | Enterococcus |
| Proteobacteria | Gammaproteobacteria | Enterobacterales | Erwiniaceae | Erwinia |
| Proteobacteria | Gammaproteobacteria | Enterobacterales | Enterobacteriaceae | Escherichia |
| Proteobacteria | Gammaproteobacteria | Enterobacteriales | Enterobacteriaceae | Escherichia/Shigella |
| Firmicutes | Clostridia | Clostridiales | Eubacteriaceae | Eubacterium |
| Proteobacteria | Gammaproteobacteria | Enterobacteriales | Enterobacteriaceae | Klebsiella |
| Firmicutes | Clostridia | Clostridiales | Lachnospiraceae | Lachnoclostridium |
| Firmicutes | Bacilli | Lactobacillales | Lactobacillaceae | Lactobacillus |
| Firmicutes | Negativicutes | Selenomonadales | Veillonellaceae | Megasphaera |
| Euryarchaeota | Methanomicrobia | Methanosarcinales | Methanosarcinaceae | Methanosarcina |
| Proteobacteria | Gammaproteobacteria | Enterobacterales | Morganellaceae | Moellerella |
| Actinobacteria | Actinobacteria | Coriobacteriales | Coriobacteriaceae | Olsenella |
| Firmicutes | Bacilli | Bacillales | Paenibacillaceae | Paenibacillus |
| Proteobacteria | Gammaproteobacteria | Enterobacteriales | Enterobacteriaceae | Pantoea |
| Proteobacteria | Gammaproteobacteria | Enterobacteriales | Enterobacteriaceae | Pectobacterium |
| Proteobacteria | Deltaproteobacteria | Desulfuromonadales | Desulfuromonadaceae | Pelobacter |
| Proteobacteria | Gammaproteobacteria | Vibrionales | Vibrionaceae | Photobacterium |
| Proteobacteria | Gammaproteobacteria | Enterobacteriales | Enterobacteriaceae | Pragia |
| Proteobacteria | Gammaproteobacteria | Enterobacteriales | Enterobacteriaceae | Proteus |
| Proteobacteria | Gammaproteobacteria | Enterobacteriales | Enterobacteriaceae | Providencia |
| Proteobacteria | Gammaproteobacteria | Enterobacteriales | Enterobacteriaceae | Raoultella |
| Proteobacteria | Gammaproteobacteria | Enterobacteriales | Enterobacteriaceae | Serratia |
| Proteobacteria | Gammaproteobacteria | Enterobacteriales | Enterobacteriaceae | Shimwellia |
| Firmicutes | Bacilli | Bacillales | Staphylococcaceae | Staphylococcus |
| Firmicutes | Bacilli | Lactobacillales | Streptococcaceae | Streptococcus |
| Firmicutes | Erysipelotrichia | Erysipelotrichales | Erysipelotrichaceae | Turicibacter |
| Proteobacteria | Gammaproteobacteria | Vibrionales | Vibrionaceae | Vibrio |
| Proteobacteria | Gammaproteobacteria | Enterobacterales | Enterobacteriaceae | Yokenella |

**Supplementary table 2. Butyrate-producing bacteria selection**

In order to assess the presence of butyrate-producing bacteria in our cohort, we measured the abundance of 17 bacterial taxa that are known to be the most abundant drivers of butyrate production, in accordance with a recently published study that analysed butyrate-producing pathways from 15 publicly available data sets [15].

| **Phylum** | **Order** | **Class** | **Family** | **Genus** |
| --- | --- | --- | --- | --- |
| Bacteroidetes | Bacteroidia | Bacteroidales | Marinifilaceae | Butyricimonas |
| Bacteroidetes | Bacteroidia | Bacteroidales | Marinifilaceae | Odoribacter |
| Bacteroidetes | Bacteroidia | Bacteroidales | Rikenellaceae | Alistipes |
| Firmicutes | Clostridia | Clostridiales | Eubacteriales | Eubacterium |
| Firmicutes | Clostridia | Clostridiales | Lachnospiraceae | Anaerostipes |
| Firmicutes | Clostridia | Clostridiales | Lachnospiraceae | Butyrivibrio |
| Firmicutes | Clostridia | Clostridiales | Lachnospiraceae | Coprococcus_2 |
| Firmicutes | Clostridia | Clostridiales | Lachnospiraceae | Coprococcus_3 |
| Firmicutes | Clostridia | Clostridiales | Lachnospiraceae | Roseburia |
| Firmicutes | Clostridia | Clostridiales | Lachnospiraceae | Shuttleworthia |
| Firmicutes | Clostridia | Clostridiales | Ruminococcaceae | Butyricicoccus |
| Firmicutes | Clostridia | Clostridiales | Ruminococcaceae | Faecalibacterium |
| Firmicutes | Clostridia | Clostridiales | Ruminococcaceae | Flavonifractor |
| Firmicutes | Clostridia | Clostridiales | Ruminococcaceae | Pseudoflavonifractor |
| Firmicutes | Clostridia | Clostridiales | Ruminococcaceae | Oscillibacter |
| Firmicutes | Clostridia | Clostridiales | Ruminococcaceae | Ruminococcus_2 |
| Firmicutes | Clostridia | Clostridiales | Ruminococcaceae | Subdoligranulum |

|  | Microbiota (n=349) | No microbiota (n=2189) | p |
| --- | --- | --- | --- |
| Age, years | 72 [62 – 80] | 74 [63 – 81] | 0.130 |
| Male sex | 194 (55.6) | 1250 (57.1) | 0.636 |
| Caucasian ethnicity | 314 (90.2) | 2052 (93.7) | 0.112 |
| **History** |  |  |  |
| Atrial fibrillation/flutter | 56 (16.0) | 339 (15.3) | 0.792 |
| Prior stroke | 109 (31.2) | 720 (32.8) | 0.596 |
| Hypertension | 208 (59.6) | 1196 (54.6) | 0.089 |
| Myocardial infarction | 44 (12.6) | 289 (13.1) | 0.857 |
| Cardiac valve disease† | 25 (6.9) | 151 (6.8) | 1.000 |
| Peripheral vascular disease | 24 (6.3) | 175 (7.7) | 0.434 |
| COPD | 30 (8.6) | 183 (8.2) | 0.860 |
| Diabetes Mellitus | 67 (19.2) | 436 (19.9) | 0.822 |
| Malignancy | 31 (8.6) | 208 (9.3) | 0.742 |
| Current smoker | 104 (29.0) | 545 (24.0) | 0.056 |
| Alcoholism | 21 (6.0) | 112 (4.6) | 0.307 |
| **Previous medication** |  |  |  |
| Anticoagulants | 37 (10.6) | 247 (11.2) | 0.793 |
| Antiplatelet therapy | 132 (37.8) | 888 (40.5) | 0.372 |
| Statins | 134 (38.4) | 817 (37.3) | 0.730 |
| Angiotensin-converting enzyme inhibitors | 114 (32.5) | 536 (24.3) | 0.001 |
| Proton pump inhibitors | 94 (26.9) | 568 (25.7) | 0.675 |
| β-blocker | 123 (35.2) | 767 (34.9) | 0.946 |
| Randomisation to Ceftriaxone | 189 (54.2) | 1079 (50.7) | 0.103 |
| **Stroke characteristics** |  |  |  |
| Cerebral infarction | 287 (82.3) | 2154 (84.0) | 0.462 |
| Transient ischaemic attack | 37 (10.6) | 232 (10.6) | 1.000 |
| Cerebral haemorrhage | 13 (3.7) | 80 (3.7) | 1.000 |
| Modified Rankin Scale score before stroke symptoms$ | 0 (0–1) | 0 (0–1) | 0.538 |
| National Institutes of Health  Stroke Scale score¶ | 5 (3-9) | 5 (3-9) | 0.312 |

**Supplementary table 3: comparison of patients participating in the microbiota sub study within the case-mix of cohort of the PASS study.**

Data are median (IQR) or n/N (%). †Cardiac valve disease was defined as cardiac valve insufficiency, stenosis, or replacement. §Scores on the modified Rankin Scale range from 0 to 6, with 6 indicating death; modified Rankin Scale scores before onset of stroke symptoms were assessed in 345 stroke patients. ¶Scores on the National Institutes of Health Stroke Scale range from 0 to 30, with 30 indicating highest degree of stroke severity; these scores were assessed in 349 patients.

**Supplementary table 4: comparison of selected plasma samples of stroke patients within the case-mix cohort of stroke patients with microbiota samples.**

|  | No plasma (n=312) | Plasma (n=32) | p |
| --- | --- | --- | --- |
| Age, years | 72 [62 – 80] | 67 [63 – 82] | 0.900 |
| Male sex | 173 (55.4) | 11 ( 34.3) | 0.157 |
| Caucasian ethnicity | 291 (90.7) | 28 ( 85.2) | 0.263 |
| **History** |  |  |  |
| Atrial fibrillation/flutter | 43 (16.5) | 4 ( 12.5) | 0.650 |
| Prior stroke | 92 (31.7) | 8 ( 25.9) | 0.687 |
| Hypertension | 184 (60.2) | 17 ( 53.1) | 0.516 |
| Myocardial infarction | 34 (13.7) | 2 (6.3) | 0.080 |
| Cardiac valve disease | 15 (4.8) | 1 (3.1) | 0.281 |
| Peripheral vascular disease | 13 (4.5) | 1 ( 3.1) | 0.862 |
| COPD | 18 (5.7) | 2 ( 6.3) | 1.000 |
| Diabetes | 56 (17.9) | 2 (6.3) | 0.061 |
| Malignancy | 19 (6.0) | 2 ( 6.3) | 1.000 |
| Current smoker | 85 (27.2) | 9 ( 28.1) | 0.766 |
| Alcoholism | 10 (3.2) | 1 ( 3.1) | 0.916 |
| **Previous medication** |  |  |  |
| Anticoagulants | 25 (8.0) | 2 ( 6.3) | 0.814 |
| Antiplatelet therapy | 117 (37.5) | 6 ( 18.8) | 0.052 |
| Statins | 119 (38.1) | 7 ( 21.9) | 0.065 |
| Angiotensin-converting enzyme inhibitors | 99 (31.7) | 6 (18.8) | 0.162 |
| Proton pump inhibitors | 79 (25.3) | 6 (18.8) | 0.423 |
| β-blocker | 108 (34.6) | 6 (18.8) | 0.092 |

**Supplementary table 5:** **Differences in beta diversity among groups**

| **Group** | **UniFrac (unweighted)** | | **UniFrac (weighted)** | | **Bray Curtis** | |
| --- | --- | --- | --- | --- | --- | --- |
|  | **p-value** | **R^2^** | **p-value** | **R^2^** | **p-value** | **R^2^** |
| Control vs Ischemic Stroke | 0.001 | 0.010 | 0.001 | 0.025 | 0.001 | 0.012 |
| Control vs Hemorraghic stroke | 0.001 | 0.048 | 0.001 | 0.098 | 0.001 | 0.036 |
| Control vs TIA | 0.614 | 0.015 | 0.373 | 0.016 | 0.580 | 0.015 |

Differences in microbiota composition among groups were tested for using permutational multivariate analysis of variance (PerMANOVA) on beta diversity matrices.

**Supplementary table 6: Lipoprotein levels of stroke patients (N=32) and controls (N=51)**

|  | Control (n=51) | Stroke patient (n=32) | p |
| --- | --- | --- | --- |
| Total Cholesterol (mg/dL) | 176.37 | 168.06 | 0.40 |
| LDL (mg/dL) | 98.60 | 98.77 | 0.97 |
| HDL (mg/dL) | 50.27 | 47.98 | 0.45 |
| Apo-A1 (mg/dL) | 133.88 | 126.89 | 0.18 |
| Apo-A2 (mg/dL) | 26.08 | 23.76 | 0.15 |
| Apo-B100 (mg/dL) | 84.59 | 83.16 | 0.69 |
| LDL/ HDL ratio | 2.00 | 2.21 | 0.67 |
| Apo B100/Apo-A1 ratio | 0.64 | 0.69 | 0.77 |

Data are displayed as mean. LDL = low-density lipoprotein; HDL = high-density lipoprotein, Apo-A = Apolipoprotein A; Apo-B = Apolipoprotein B.

**Supplementary table 7: Logistic regression on role of butyrate-producing bacteria on risk of infection in patients without ceftriaxone exposure (n=163)**

|  | **Univariate** | | **Multivariate** | |
| --- | --- | --- | --- | --- |
|  | Odds ratio [2.5% – 97.5%] | p-value | Odds ratio [2.5% – 97.5%] | p-value |
| Age | 1.04 [0.97–1.10] | 0.183 |  |  |
| Male sex | 0.75 [0.21–2.61] | 0.653 |  |  |
| Diabetes | 3.04 [0.75–10.97] | 0.09 | 3.13 [0.68– 12.88] | 0.117 |
| Prior stroke | 0.93 [0.20–3.39] | 0.915 |  |  |
| NIHSS > 10 | 2.27 [0.56–8.06] | 0.216 |  |  |
| Butyrate-producing bacteria  (log abundance) | 0.70 [0.53–0.90] | 0.007 | 0.70 [0.51– 0.91] | 0.013 |

**Supplementary figure 1:** Flowchart of study inclusions

2165 patients not included in microbiota sub study

2550 patients enrolled

12 patients withdrew consent directly after randomisation

2538 patients analysed

24 patients lost to follow-up

2514 patients completed follow up

349 patients included in microbiota sub study


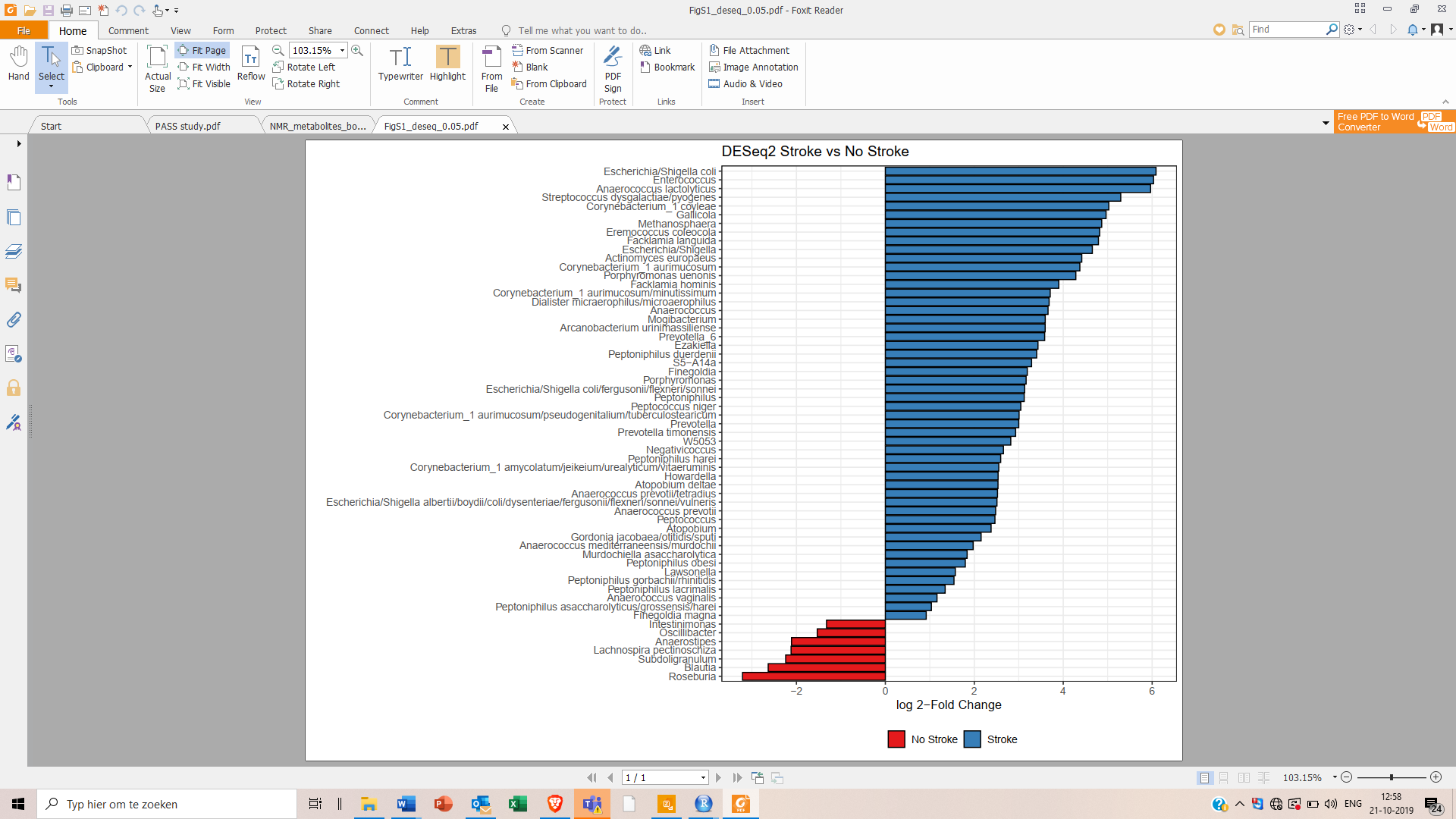
**Supplementary figure 2**: **DESeq2 of significant differences (corrected for multiple testing by Benjamini-Hochberg) of individual microbial taxa** **between stroke patients (n=349; blue) and age- and sex-matched controls (n=51; red)**

**Supplementary figure 3: No differences in Firmicutes to Bacteroidetes ratio between stroke patients and controls**


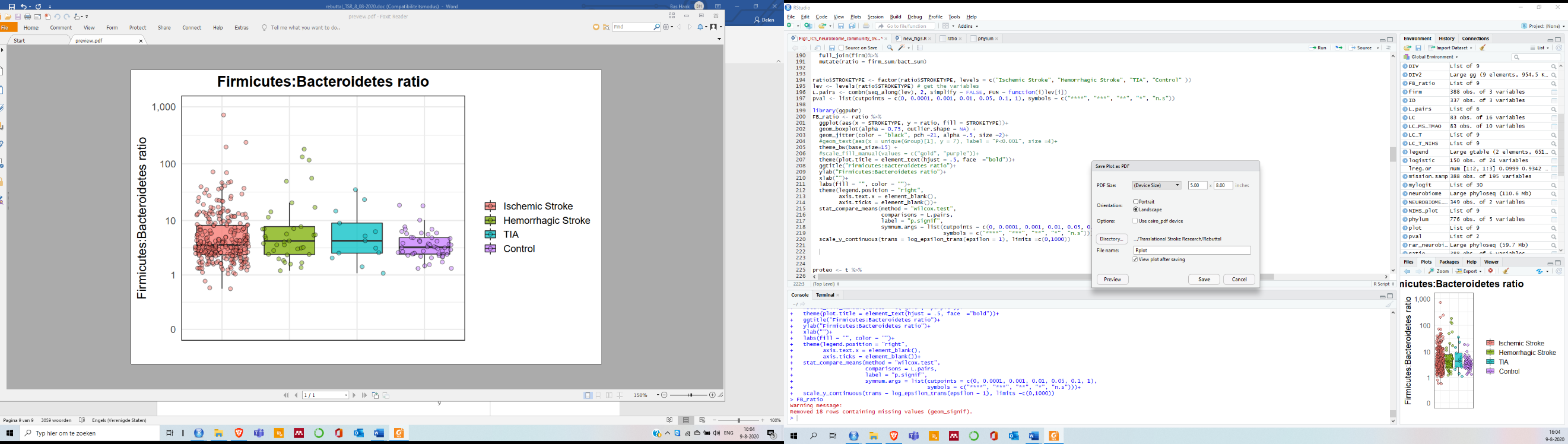


Data are presented as box plot overlaid by a dot plot with a line at the median.


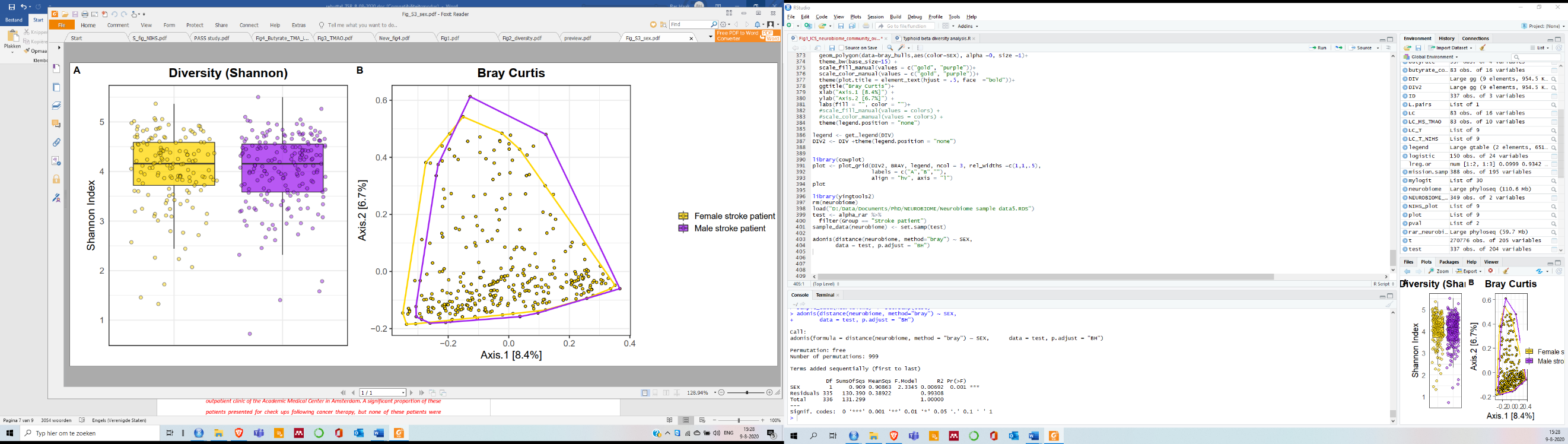
**Supplementary figure 4: No differences in alpha and beta diversity between male (n=194) and female patients (n=155) with stroke.**

*R^2^*=0.007; *p*=0.136

(A) The Shannon index was used to calculate the alpha diversity community within each individual microbiota sample. Data are presented as box plot overlaid by a dot plot with a line at the median. (B) Beta diversity as depicted by Bray-Curtis dissimilarity index in a PCoA representation.


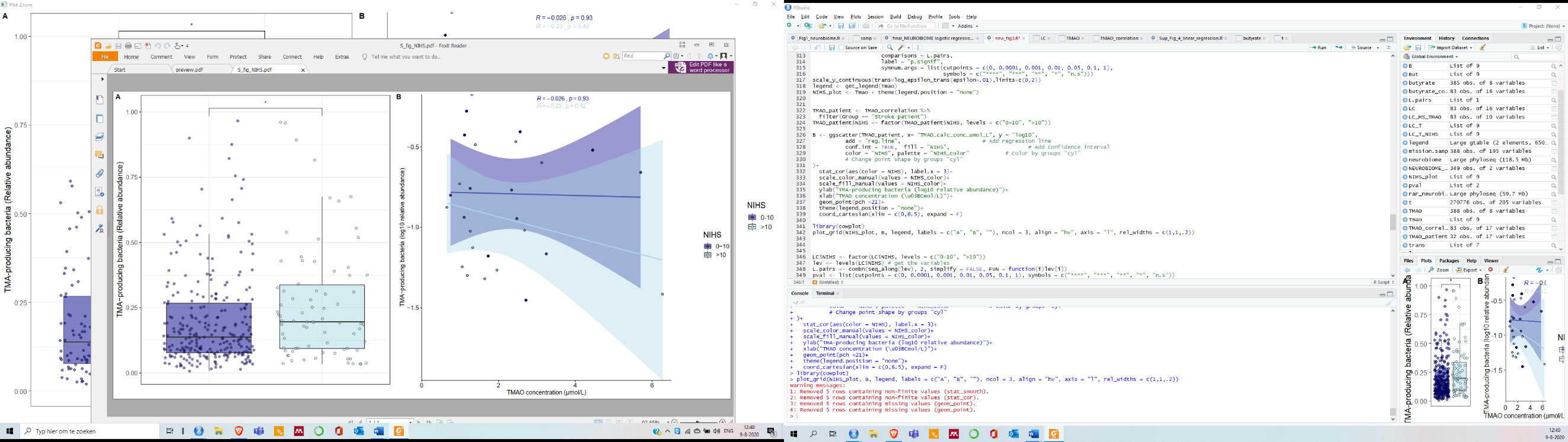
 **Supplementary figure 5: Increased abundance of TMA-producing bacteria depending on stroke severity.**

(A) Data are presented as box plot overlaid by a dot plot with a line at the median. (B) Scatter plot of TMAO concentrations umol/L, x-axis) versus relative abundance of TMA-producing bacteria (percentage after log10 transformation, y-axis). The line represents the linear regression fit stratified by NIHS score (tested for linearity with Wald tests) and the shade the corresponding 95% confidence interval. Corresponding Pearson correlation coefficients and p-values are also displayed in the figure. Severe stroke is determined by the National Institute of Health Stroke (NIHS) scale >10 (N= 97 patients); mild stroke is determined by a NIHS scale <10 (N=252 patients). * = p<0.05

**Supplementary figure 6: Unsupervised clustering between stroke and no stroke patients based on NMR profiles**


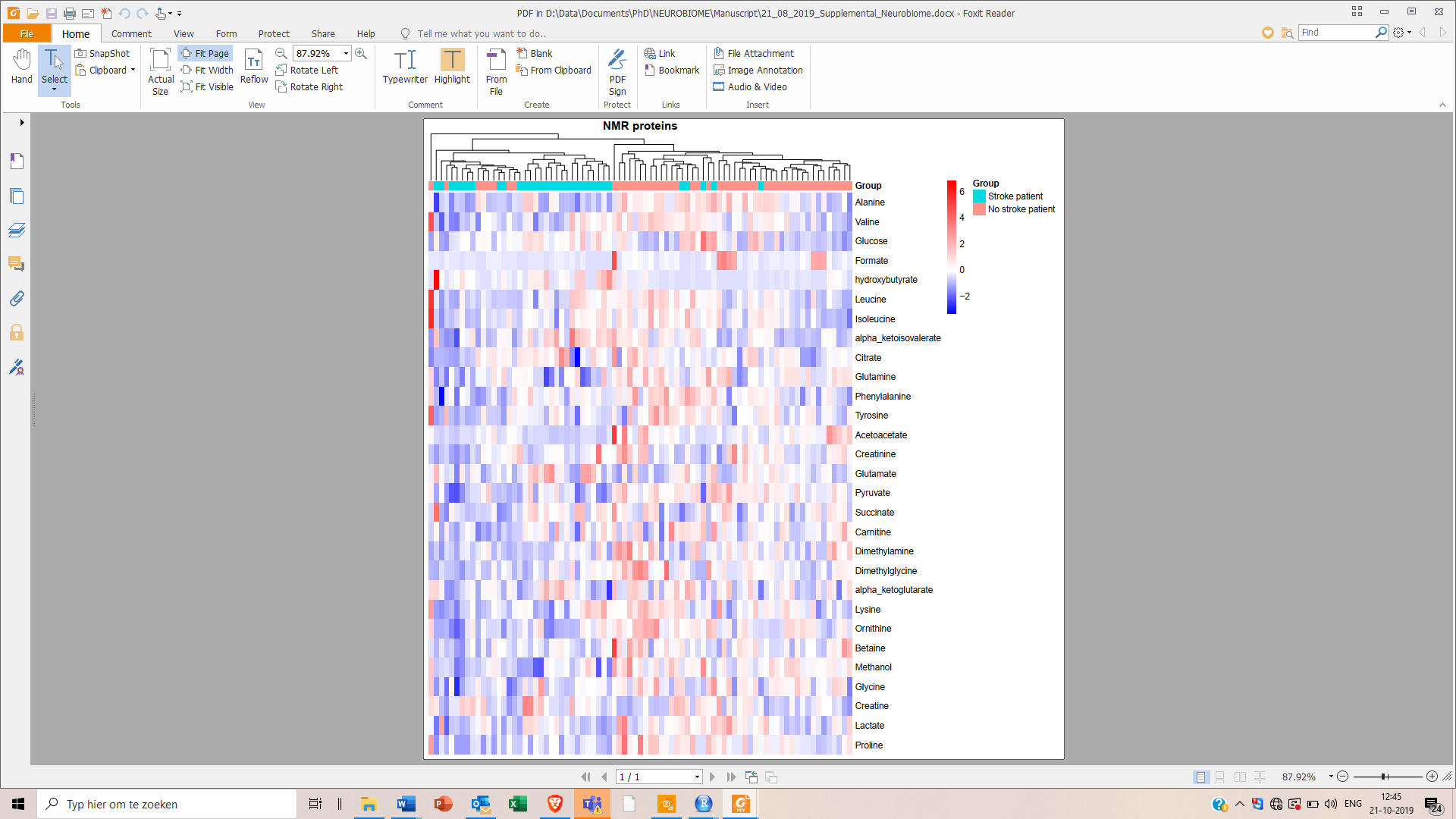


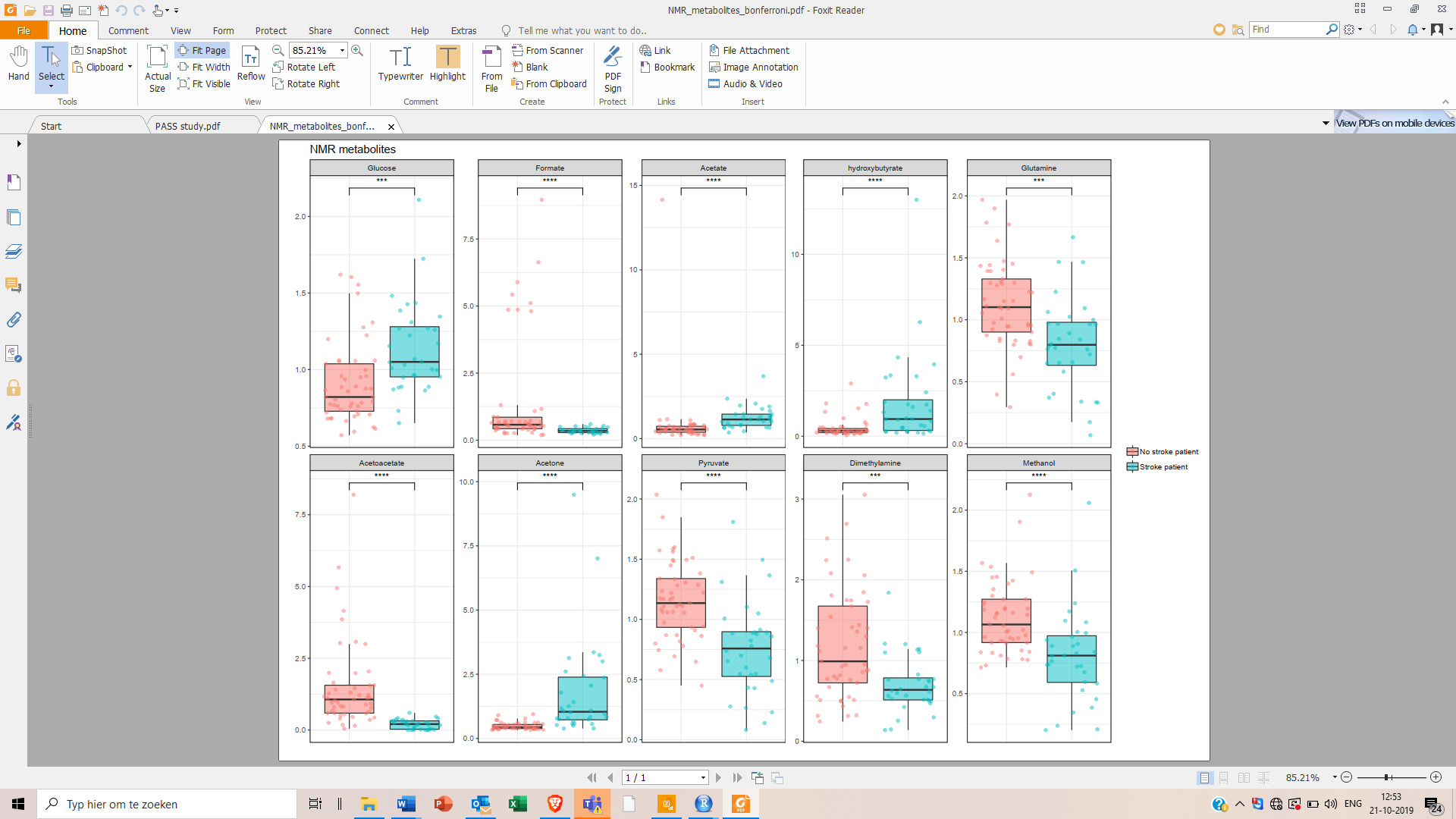

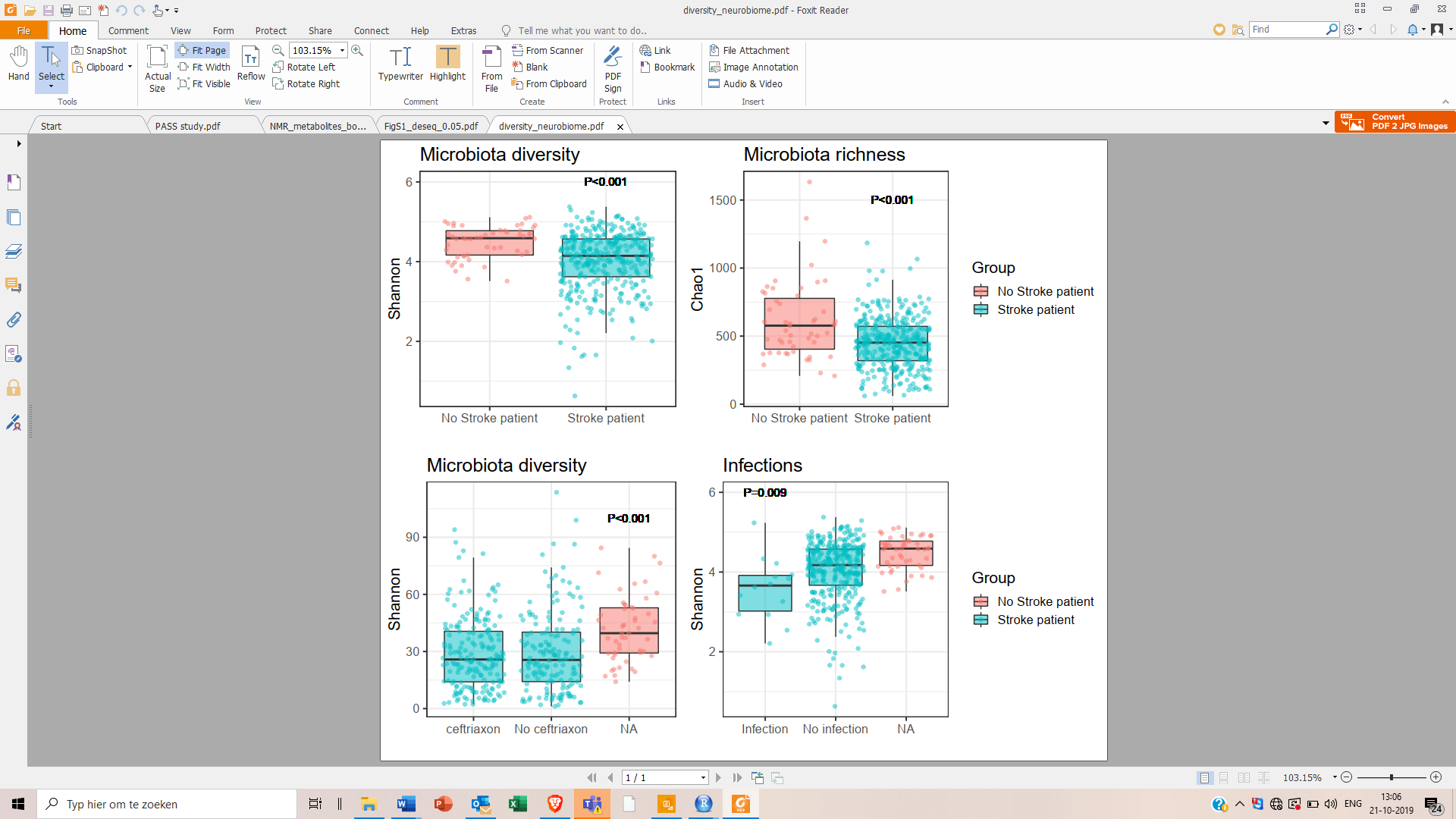
**Supplementary figure 7. Overview of metabolites between stroke patients and as characterized by NMR.** Data are presented as box plot overlaid by a dot plot with a line at the median. *** = p <0.001, **** = p<0.0001


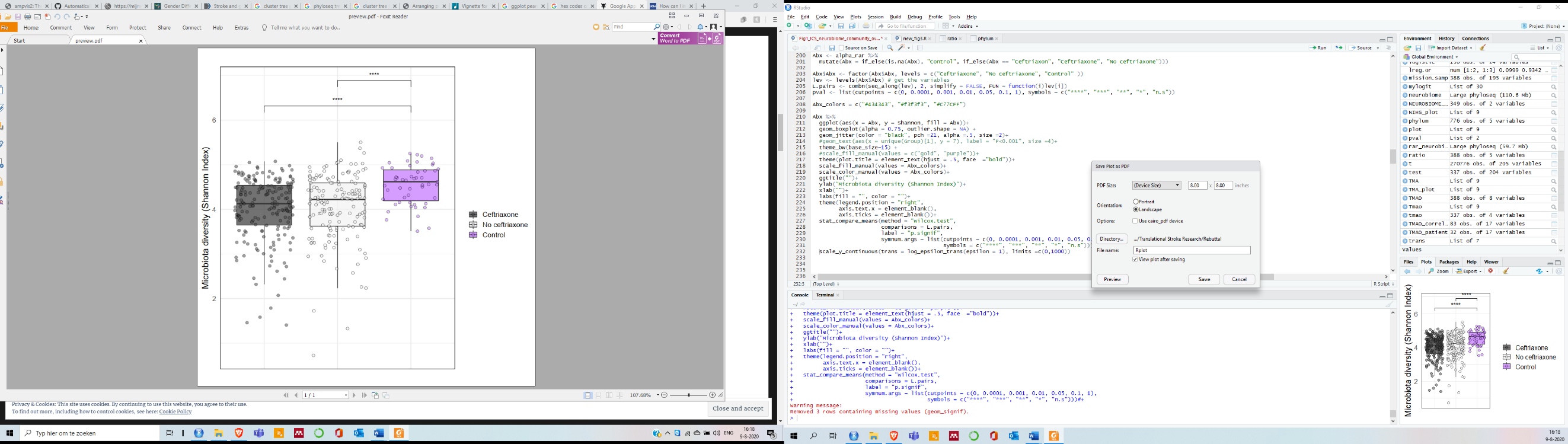
**Supplementary figure 8: Effect of ceftriaxone exposure on alpha diversity of gut microbiota.**

Data are presented as box plot overlaid by a dot plot with a line at the median. **** = p<0.0001

**References**

1. Yu Z, Morrison M. Improved extraction of PCR-quality community DNA from digesta and fecal samples. Biotechniques. 2004;36:808–12.

2. Costea PI, Zeller G, Sunagawa S, Pelletier E, Alberti A, Levenez F, et al. Towards standards for human fecal sample processing in metagenomic studies. Nat Biotechnol. 2017;35:1069–76.

3. Kozich JJ, Westcott SL, Baxter NT, Highlander SK, Schloss PD. Development of a Dual-Index Sequencing Strategy and Curation Pipeline for Analyzing Amplicon Sequence Data on the MiSeq Illumina Sequencing Platform. Appl Environ Microbiol. 2013;79:5112–20.

4. Edgar RC. Search and clustering orders of magnitude faster than BLAST. Bioinformatics. 2010;26:2460–1.

5. Callahan BJ, McMurdie PJ, Rosen MJ, Han AW, Johnson AJA, Holmes SP. DADA2: High-resolution sample inference from Illumina amplicon data. Nat Methods. 2016;13:581–3.

6. Wang Q, Garrity GM, Tiedje JM, Cole JR. Naive Bayesian Classifier for Rapid Assignment of rRNA Sequences into the New Bacterial Taxonomy. Appl Environ Microbiol. 2007;73:5261–7.

7. Quast C, Pruesse E, Yilmaz P, Gerken J, Schweer T, Yarza P, et al. The SILVA ribosomal RNA gene database project: Improved data processing and web-based tools. Nucleic Acids Res. 2013;41:590–6.

8. Findeisen M, Brand T, Berger S. A1H-NMR thermometer suitable for cryoprobes. Magn Reson Chem. 2007;45:175–8.

9. Price WS. Water Signal Suppression in NMR Spectroscopy. Annu Reports NMR Spectrosc. 1999;28:289–354.

10. Wu PSC, Otting G. Rapid pulse length determination in high-resolution NMR. J Magn Reson. 2005;176:115–9.

11. Wu DH, Chen AD, Johnson CS. An Improved Diffusion-Ordered Spectroscopy Experiment Incorporating Bipolar-Gradient Pulses. J Magn Reson Ser A. 1995;115:260–4.

12. Kumar A, Ernst RR, Wüthrich K. A two-dimensional nuclear Overhauser enhancement (2D NOE) experiment for the elucidation of complete proton-proton cross-relaxation networks in biological macromolecules. Biochem Biophys Res Commun. 1980;95:1–6.

13. Verhoeven A, Giera M, Mayboroda OA. KIMBLE: A versatile visual NMR metabolomics workbench in KNIME. Anal Chim Acta. 2018;1044:66–76.

14. Rath S, Heidrich B, Pieper DH, Vital M. Uncovering the trimethylamine-producing bacteria of the human gut microbiota. Microbiome. Microbiome; 2017;5:1–14.

15. Vital M, Karch A, Pieper DH. Colonic Butyrate-Producing Communities in Humans: an Overview Using Omics Data. Shade A, editor. mSystems. 2017;2:43–51.
